# Proteomic Selection of Immunodiagnostic Antigens for Human African Trypanosomiasis and Generation of a Prototype Lateral Flow Immunodiagnostic Device

**DOI:** 10.1371/journal.pntd.0002087

**Published:** 2013-02-28

**Authors:** Lauren Sullivan, Steven J. Wall, Mark Carrington, Michael A. J. Ferguson

**Affiliations:** 1 College of Life Sciences, University of Dundee, Dundee, United Kingdom; 2 BBInternational, Alchemy House, Dundee, United Kingdom; 3 Department of Biochemistry, University of Cambridge, Cambridge, United Kingdom; Yale School of Public Health, United States of America

## Abstract

**Background:**

The diagnosis of Human African Trypanosomiasis relies mainly on the Card Agglutination Test for Trypanosomiasis (CATT). While this test is successful, it is acknowledged that there may be room for improvement. Our aim was to develop a prototype lateral flow test based on the detection of antibodies to trypanosome antigens.

**Methodology/Principal Findings:**

We took a non-biased approach to identify potential immunodiagnostic parasite protein antigens. The IgG fractions from the sera from *Trypanosoma brucei gambiense* infected and control patients were isolated using protein-G affinity chromatography and then immobilized on Sepharose beads. The IgG-beads were incubated with detergent lysates of trypanosomes and those proteins that bound were identified by mass spectrometry-based proteomic methods. This approach provided a list of twenty-four trypanosome proteins that selectively bound to the infection IgG fraction and that might, therefore, be considered as immunodiagnostic antigens. We selected four antigens from this list (ISG64, ISG65, ISG75 and GRESAG4) and performed protein expression trials in *E. coli* with twelve constructs. Seven soluble recombinant protein products (three for ISG64, two for ISG65 and one each for ISG75 and GRESAG4) were obtained and assessed for their immunodiagnostic potential by ELISA using individual and/or pooled patient sera. The ISG65 and ISG64 construct ELISAs performed well with respect to detecting *T. b. gambiense* infections, though less well for detecting *T. b. rhodesiense* infections, and the best performing ISG65 construct was used to develop a prototype lateral flow diagnostic device.

**Conclusions/Significance:**

Using a panel of eighty randomized *T. b. gambiense* infection and control sera, the prototype showed reasonable sensitivity (88%) and specificity (93%) using visual readout in detecting *T. b. gambiense* infections. These results provide encouragement to further develop and optimize the lateral flow device for clinical use.

## Author Summary

Human African Trypanosomiasis is caused by infection with *Trypanosoma brucei gambiense* or *T. b. rhodesiense*. Preliminary diagnosis of *T. b. gambiense* infection relies mainly on a Card Agglutination Test for Trypanosomiasis (CATT), which has acknowledged limitations. New approaches are needed, first to identify new diagnostic antigens and, second, to find a more suitable platform for field-based immunodiagnostic tests. We took an unbiased approach to identify candidate diagnostic antigens by asking which parasite proteins bind to the antibodies of infected patients and not to the antibodies of uninfected patients. From this list of twenty-four candidate antigens, we selected four and from these we selected the one that worked the best in conventional immunodiagnostic tests. This antigen, ISG65, was used to make lateral flow devices, where a small sample of patient serum is added to a pad and thirty minutes later infection can be inferred by simple optical read out. This simple prototype device works as well as the CATT test and may be developed and optimized for clinical use in the field.

## Introduction

Human African Trypanosomiasis (HAT), also known as Sleeping Sickness, is a disease caused by *Trypanosoma brucei gambiense* and *T. b. rhodesiense* [1], [2], [3]. The parasites are transmitted in sub-Saharan Africa by the bite from an infected tsetse fly. HAT is of great public health significance, with epidemic outbreaks recorded several times over the past century with, at times, estimates of 300,000 or more infected individuals [4]. Today, the recorded number of new cases has dropped below 10,000 per year, yet HAT still continues to place a large burden on individuals and communities in terms of disability-adjusted life years [5], [6]. The identification of infected individuals is crucial for therapeutic and public health intervention. New tools could aid eradication of this disease when used in coordination with other efforts [5], [7], [8].

Infection with *T. b. gambiense* or *T. b. rhodesiense* progresses through two defined stages. The first stage is when trypanosomes are limited to the blood and lymphatic systems. The second stage occurs when the parasites invade the central nervous system [2]. The latter leads to neurological damage, sleep cycle disruption, coma and death if the patient does not receive treatment [9], [10], [11]. The two stages are treated with different drugs, and those used for the second stage have severe toxic side effects [12], [13]. Staging of the infection, to select the appropriate therapeutics and follow up, is currently done by sampling the cerebral spinal fluid to search for the presence of parasites and/or increased numbers of lymphocytes [14]. The view that human trypanosome infections are invariably fatal if not treated has been challenged recently [15], [16] but, nevertheless, early diagnosis is extremely important both for individual patient outcomes and for controlling epidemic spread [17], [18].

The identification of infected individuals relies on dedicated screening teams that visit at-risk communities or patients seeking medical examination [19]. HAT diagnosis in the field faces many difficulties; not least the logistical challenges for the screening teams to attend communities in rural locations. In endemic areas, civil disturbance usually increases the incidence of HAT and decreases the frequency of screening [20], [21], [22]. Once the screening teams are with the communities, they face further challenges to recruit the entire local population into the HAT screening programme, which can lead to under-reporting and under-estimations of infection rates [23], [24], [25], [26].

The current HAT screening regimen uses the Card Agglutination Test for Trypanosomiasis (CATT), a serological test that detects whether antibodies from an individual are able to aggregate a suspension of fixed and stained *T. b. gambiense* trypanosomes [27], detecting primarily antibodies to the variant surface glycoproteins (VSGs) on the fixed cells. If patients have a positive CATT result, microscopic examination of their blood is carried out to detect trypanosomes. If this is positive, a lumbar puncture is performed to stage the infection. Over the years, the CATT test has been optimised to improve sensitivity, specificity and stability. Such modifications include dilution of the blood samples, the use of multiple trypanosome clones expressing different VSG variants and improvements in thermostability [28], [29], [30], [31]. Despite the usefulness and wide deployment of the CATT test, it has several widely accepted limitations [32], [33], [34], [35]. These include varying degrees of sensitivity and specificity, its inability to detect *T. b. rhodesiense* infections, the requirement for trained screening personnel to use it and the specialised manufacture which precludes production on a scale necessary to saturate the market [26], [36]. There have also been other post-CATT test diagnostic enhancements. For example, the concentration of trypanosomes from infected blood to improve microscopic detection [37], [38], [39]. Further, the detection of trypanosome DNA in blood by loop-mediated isothermal amplification of DNA (LAMP) [40] methods are under investigation and are summarised in a recent review [41]. However, these diagnostic methods require relatively sophisticated laboratory equipment. In summary, there is well accepted case for developing an extremely simple, low-cost diagnostic device with greater sensitivity and specificity than current field tests [4].

Lateral flow devices are simple tests that can rapidly detect nanogram amounts of antibodies or antigens in finger-prick blood samples without the need for any ancillary equipment [42]. *T. b. gambiense* infections are characterised by very low parasitemias, often <1000/ml, the equivalent of <5 ng total trypanosome protein/ml blood. Thus, using currently available technology, it is not feasible to directly detect a trypanosome protein and a lateral flow test that detects host antibodies is perhaps more likely to have the required sensitivity and specificity. The manufacture of large numbers of lateral flow devices requires milligram to gram amounts of diagnostic antigen, therefore potential diagnostic antigens for such devices should preferably derive from recombinant or synthetic sources. Recently the Foundation for Innovative New Diagnostics (FIND) has invested in developing new diagnostic tests for human African Trypanosomiasis [43]. With a similar aim in mind, we also set out to identify novel diagnostic antigens, and to create a prototype lateral flow test device, but using a non-biased (proteomics) approach to select potential biomarker antigens. The results of this antigen selection and the performance of a prototype lateral flow device are reported here.

## Materials and Methods

### Ethics statement

All human serum samples were collected with the informed consent of the patients that they could be used anonymously for diagnostic development. Rodents were used to propagate sufficient *T. brucei* parasites to make the detergent lysates for immunoaffinity chromatography and proteomics. The animal procedures were carried out according the United Kingdom Animals (Scientific Procedures) Act 1986 and according to specific protocols approved by The University of Dundee Ethics Committee and as defined and approved in the UK Home Office Project License PPL 60/3836 held by MAJF.

### Serum samples and storage

Two sets of human serum samples were used, the first was kindly provided by Philippe Büscher (Institute of Tropical Medicine, Antwerp) and consisted of nine sera from *T. b. gambiense* infected patients and nine from matched non-infected patients. These samples underwent virus inactivation using a procedure that retains antibody reactivity [44]. Briefly, 1% Tri(*n*-butyl)phosphate (TnBP) and 1% Triton X-45 were added to thawed serum samples and incubated at 31°C for 4 h. Sterile castor oil was added, mixed and the samples were centrifuged (3800× *g*, 30 min). The oil-extraction was repeated three times and the virus-inactivated sera (lower phases) were aliquoted and stored at −80°C. The second set of 145 patient sera (200 µl aliquots) was obtained from the WHO Human African Trypanosomiasis specimen bank [45]. Serum samples were aliquoted and stored at either −80°C for long-term storage or in 50% glycerol at −20°C when prepared for ELISA analysis. Freeze-thawing was kept to a minimum; samples from P. Büscher and WHO were freeze-thawed three times and twice, respectively, prior to use in ELISA tests.

### IgG purification from serum

Following virus inactivation, 125 µl of sera from four infected and four uninfected (control) patients were pooled. Each pool was applied to a 1 ml protein G column (GE Healthcare) equilibrated in phosphate buffered saline (PBS). The columns were washed with 10 ml of PBS and the bound IgG antibodies were eluted with 50 mM sodium citrate pH 2.8, and collected in 1 ml fractions into tubes containing 200 µl of 1 M Tris-HCl, buffer pH 8.5. Peak fractions containing IgG were combined and dialysed for 16 h against coupling buffer (0.1 M NaHCO_3_, 0.5 M NaCl, pH 8.3).

### Coupling of IgG to CNBr-activated Sepharose

CNBr-activated Sepharose (GE Healthcare) was hydrated in 1 mM HCl and then equilibrated in coupling buffer. Aliquots (0.75 ml packed volume) were mixed with 7.2 mg of purified infection IgG or purified control IgG in a final volume of 3 ml coupling buffer for 16 h at 4°C. The coupling of IgG was confirmed by measuring the absorbance of the supernatant at 280 nm before and after coupling. The Sepharose-IgG conjugates were centrifuged at 500× *g* (10 mins, 4°C) and the beads were resuspended in 15 ml 1 M ethanoamine, pH 9, to block remaining amine-reactive sites for 2 h at room temperature. Following this, the IgG-Sepharose beads were washed with three cycles of 0.1 M Tris-HCl, pH 8.0, 0.5 M NaCl followed by 0.1 M sodium acetate buffer, pH 6.0, 0.5 M NaCl and finally washed and stored in PBS containing 0.05% NaN_3_.

### Preparation *T. b. brucei* lysate

Six BalbC mice were injected with *T. b. brucei* Lister 427 variant MITat 1.4 cells. After three days, infected mouse blood was harvested with citrate anticoagulant, adjusted to 10^7^ parasites per ml with PBS and aliquots of 0.5 ml were injected into the peritoneal cavity of 12 Wistar rats. The rat blood was harvested after 3 days with citrate anticoagulant and centrifuged at 1000× *g* for 10 min at 4°C. Plasma was removed and the buffy layer was resuspended in separation buffer plus glucose (SB + glucose; 57 mM Na_2_HPO_4_, 3 mM KH_2_PO_4_, 44 mM NaCl, 10 g/l glucose) and applied to a DE52 DEAE-cellulose (Whatman) column that had been pre-equilibrated with SB + glucose. The trypanosomes were washed through the column with SB + glucose, counted, centrifuged (900 g, 15 min, 4°C), resuspended in 1 ml PBS and then adjusted to 1×10^9^ parasites/ml in ice-cold lysis buffer (50 mM Na_2_PO_4_, pH 7.2, 2% *n*-octyl β-D-glucopyranoside (nOG) detergent, 1 mM PMSF, 1 mM TLCK, 1 µg/ml aprotinin, 1 µg/ml leupeptin and 1× Roche protease cocktail minus EDTA). The lysate was incubated for 30 min on ice and then centrifuged at 100,000 *g* for 1 h at 4°C.

### Immunoprecipitation

Aliquots of *T. b. brucei* lysate (10^10^ cell equivalents) were incubated with 0.75 ml packed volume of each of the Sepharose-IgG (infection and non-infection/control) gels, rotating for 3 h at 4°C. The gels were then packed into disposable 10 ml columns and washed with 10 ml of 10 mM Na_2_PO_4_, pH 7.2, 200 mM NaCl, 1% nOG, followed by 10 ml of 5 mM Na_2_PO_4_ pH 7.2, 1% nOG. The trypanosome proteins were eluted 3 times with 750 µl of 250 mM sodium citrate, pH 2.8, 1% nOG and the eluates were pooled and neutralised with 1.5 M Tris-HCl, pH 9 and further concentrated to 140 µl using a centrifugal concentrator (Millipore, 0.5 ml capacity with 3 kDa MW cut off membrane). To remove eluted IgG, this fraction was mixed with 30 µl PBS-equilibrated Protein G agarose beads (Pierce) and incubated for 10 min and removed by centrifugation. The supernatant, containing the trypanosome proteins, were then transferred to low binding Eppendorf tubes and the proteins were precipitated by adding 1 ml ice-cold ethanol and incubation for 34 h at −20°C.

### Proteomic protein identification

Following ethanol precipitation, the proteins eluted from the infection IgG and control IgG gels were dissolved in SDS sample buffer, reduced with DTT and run on a precast 4–12% BisTris gradient SDS-PAGE gel (Invitrogen) using the MES running system. The gel was stained with colloidal Coomassie blue and equivalent regions of the infection and control lanes were cut out, reduced and alkylated with iodoacetamide and digested in-gel with trypsin. The tryptic peptides were analysed by LC-MS/MS on a Thermo Orbotrap XL system and MASCOT software was used to match peptides to the predicted trypanosome protein databases (combined GeneDB and UniProt predicted protein sequences).

### Selection of antigens and the cloning and sequencing of antigen gene segments

Trypanosome proteins identified uniquely in the infection IgG immunopurified fractions were considered for recombinant expression. Within these, proteins with high MASCOT scores, likely to be the most abundant, were prioritised for recombinant expression and purification trials. These proteins included Gene Related to Expression Site Associated Gene (GRESAG) 4, Invariant Surface Glycoprotein (ISG) 75, ISG65 and ISG64. The identified protein sequences were used to BLAST search the *T. b. brucei* predicted protein database, revealing several related protein sequences in each family. CLUSTALW2 alignments were carried out in order to better understand sequence sub-groups within those protein families. Representative gene segments from each protein sub-group that contained the peptide sequences identified by mass spectrometry were amplified from EATRO1125 genomic DNA (for ISG65-1, ISG65-2, ISG64-2, ISG64-3 and ISG75-1) or from stain 427 genomic DNA (for ISG64-1 and GRESAG4) by PCR using the primers described in the Supporting Information (Table S1). In each case, the products of three separate PCR reactions were cloned into a TOPO-TA vector (pCR2.1) for sequencing (DNA Sequencing Service, College of Life Sciences, University of Dundee).

### Recombinant protein expression and purification

**Figure 1:**
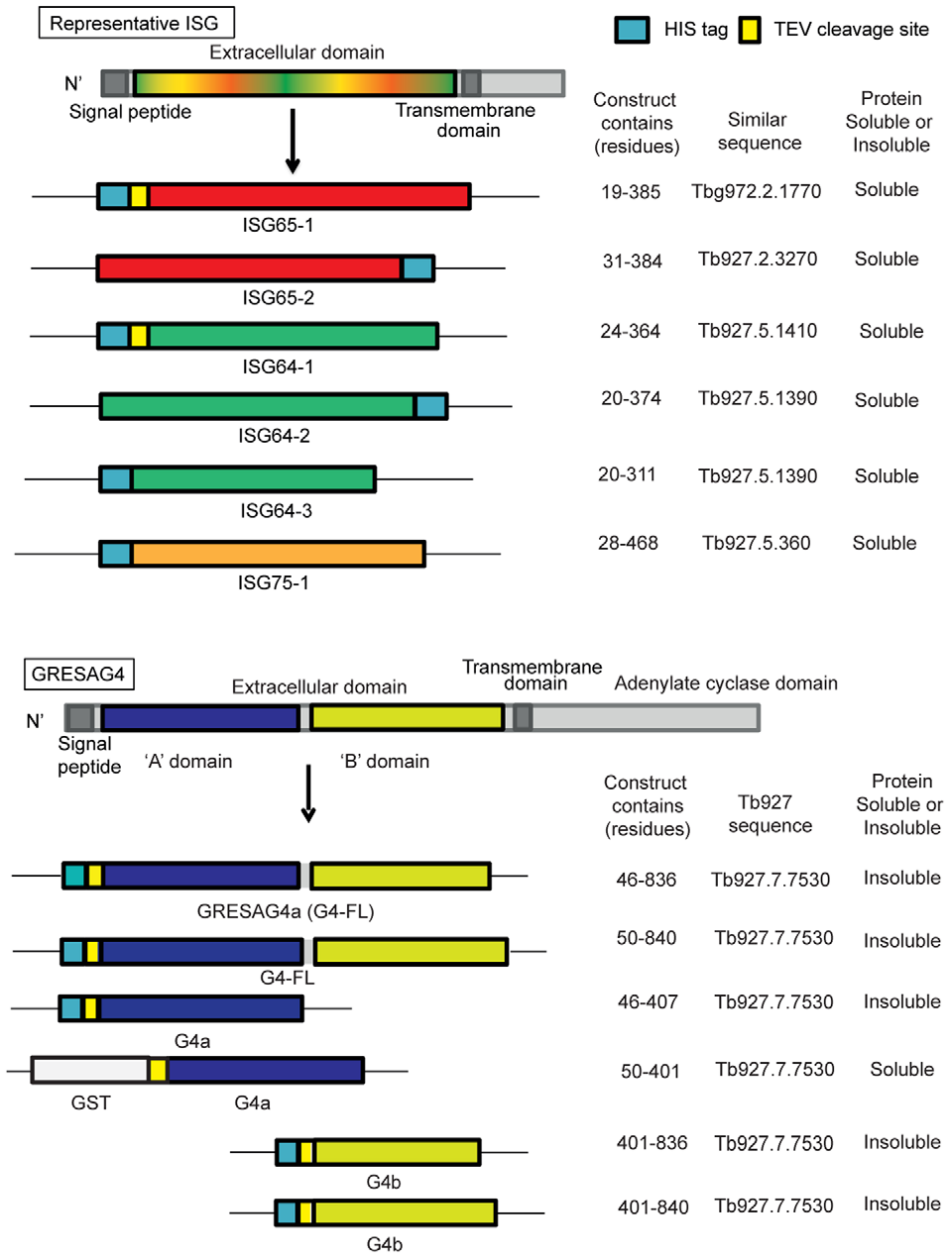
Recombinant protein antigens used in this study. A generic representation of the ISGs is shown at the top and a representation of GRESAG4 is shown at the bottom. All have cleavable N-terminal signal peptides and internal transmembrane domains, typical of type-1 membrane proteins. The constructs prepared and expressed and the soluble proteins successfully purified, are indicated.

The amplified ISG gene segments were cloned into various pET bacterial expression plasmids that provide a His-tag fused either to the N-terminus or C-terminus of the protein, in some cases via a TEV protease cleavage site, as indicated in (Figure 1). Multiple constructs were designed for GRESAG4 encoding the predicted full-length extracellular domain and several small globular domains based on predictions from GLOBplot software [46] (Figure 1). These constructs were amplified from genomic DNA using the primers described in the Supporting Information (Table S1), cloned into TOPO-TA vector pCR2.1 and verified by DNA sequencing. The constructs were either cloned into the pET15bTEV vector, such that the proteins they encode are fused at the N-terminus to a His tag, or into a pGEX-TEV vector such that the protein is fused at its N-terminus to a glutathione S-transferase (GST) sequence via a TEV cleavage site (Figure 1). The details of protein expression in *E. coli* and subsequent purification are described in the Supporting Information (Text S1).

### Enzyme-linked immunosorbent assays (ELISA)

White (Costar) un-treated 96 well plates were coated at 50 µl/well for 16 h at 4°C with 2 µg/ml recombinant protein diluted in plating buffer (0.05 M NaHCO_3_, pH 9.6). Plating solution was removed and wells were blocked with PBS containing 5% BSA, 200 µl/well for 3 h at 22°C or 16 h at 4°C. Plates were stored at 4°C and used within 24 h. Aliquots (50 µl) of serial serum dilutions (see below) were transferred in triplicate by a liquid handling device (Bio-Tek, Precision) to the ELISA plates and incubated for 1 h at room temperature, aspirated and 150 µl ELISA wash buffer was added to each well by the liquid handling device, left for 10 min and aspirated. This wash cycle was performed three times. Biotinylated goat anti-human-IgG (Jackson Immunoresearch) was diluted to 1∶5000 and 50 µl aliquots were applied to each well. After 1 h incubation at room temperature the secondary antibody solution was removed and wells were washed three times, as described above. Horseradish peroxidase (HRP) conjugated to NeutrAvidin (Sigma) was diluted to 1∶4000 and applied to the wells (50 µl/well) for 1 h at room temperature. Wells were washed as before. Finally, chemiluminescent Femto substrate (Pierce) diluted 1∶5 (*i.e.*, 0.5 ml solution A, 0.5 ml solution B with 4 ml PBS) was applied to the wells at 50 µl/well and plates were read using an Envision plate reader after 2.5 min incubation at 22°C.

ELISA measurements were made with both pooled and individual serum samples. Serum pools were made by combining patients sera from; stage 1 *T. b. gambiense* patients (n = 10), stage 2 *T. b. gambiense* patients (n = 40) and matched uninfected patients (n = 50); and from stage 1 *T. b. rhodesiense* patients (n = 5), stage 2 *T. b. rhodesiense* patients (n = 20) and matched uninfected patients (n = 25). The pooled sera were diluted to 1∶60 in 50% glycerol, PBS and 1% BSA and stored at −20°C. For ELISA assays, the 1∶60 diluted pooled sera were further diluted to 1∶1000 in PBS, 0.1% BSA and then serially diluted (doubling dilutions) to 1∶32,000. For the individual sera, the 1∶60 diluted samples were further diluted to 1∶1000 immediately before use.

### Randomisation of sera

Sera were randomised by a member of the University of Dundee Tissue Bank. Forty *T. b. gambiense* infected patients sera and forty *T. b. gambiense* uninfected patients sera were randomly selected from the fifty *T. b. gambiense* infected and fifty uninfected WHO patient sera. These eighty serum samples were then randomised and coded.

### Prototype lateral flow test

Serum aliquots (5 µl) were diluted with 15 µl PBS and applied to the sample pad. Chase buffer (80 µl of PBS, 0.05% Tween 20) was added to the sample pad and the test was allowed to develop for 30 min. The test line was visually scored and the device was opened and the sample pads (at top and bottom of nitrocellulose membrane) were removed to prevent backflow. The lateral flow tests were photographed and scanned using a densitometer (CAMAG TLC scanner 3, CAMAG).

### Statistics

Bar graphs and scatter plots (x by y) were generated by Microsoft Excel. Box plots, Receiver Operator Characteristic (ROC) curves, antigen scatter plots (y axis only) were generated by SigmaPlot 12. Statistical analysis included Mann-Whitney (Rank Sum Test) and Dunn's post-hoc (Analysis of Variance (ANOVA) on rank) in SigmaPlot 12. Data were tested for normality by Kolmogorov-Smirnov test and were further processed by Mann-Whitney or Dunn's post-hoc tests. The P values were recorded for Mann-Whitney with <0.05 set as the cut off for statistical significance.

## Results

### Antigen identification

**Figure 2:**
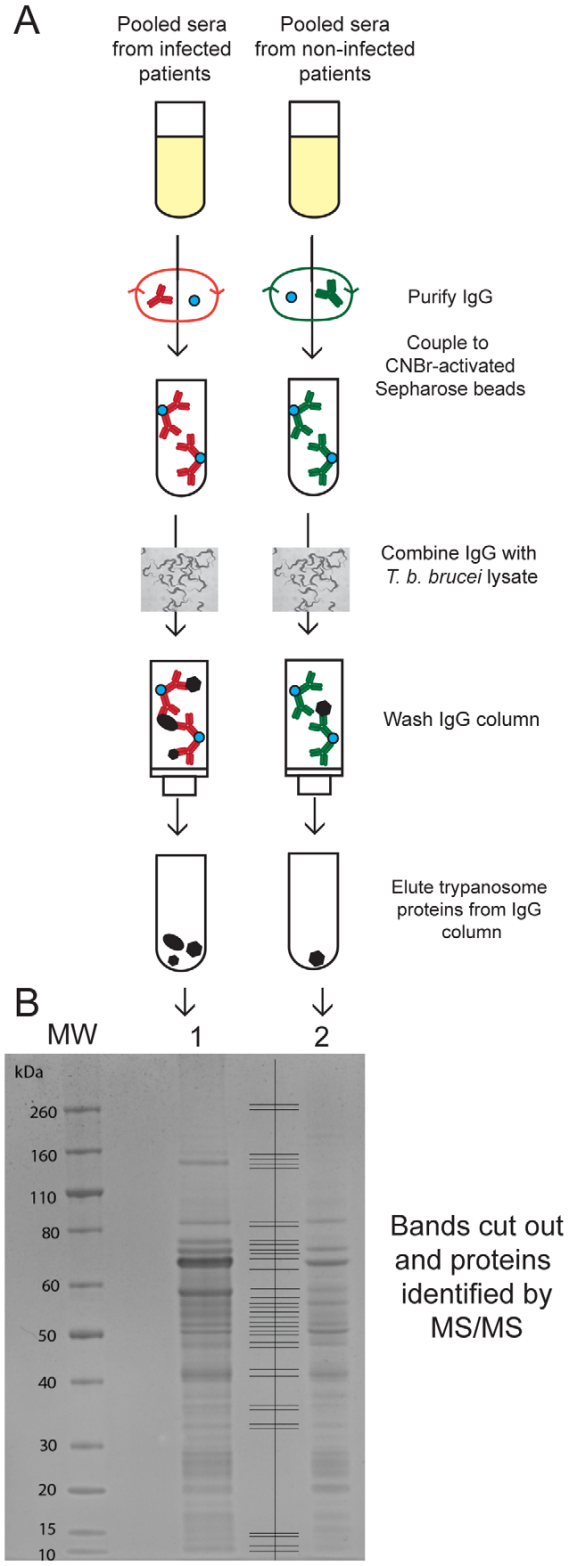
Immuno-affinity chromatography and identification of potential diagnostic antigens. (A) Schematic representation of the preparation of IgG-Sepharose from *T. b. gambiense* infection and non-infection (control) sera, the immune-affinity capture of trypanosome antigens from a whole detergent lysate and their subsequent elution and concentration by ethanol precipitation. (B) Colloidal Comassie blue stained SDS-PAGE gel of the proteins eluted from infection IgG-Sepharose (lane 1) and non-infection (control) IgG-Sepharose (lane 2). The gel lanes were excised in 18 slices per lane, as indicated between lanes 1 and 2, and analysed by LC-MS/MS after reduction, S-alkylation and tryptic digestion. The positions of molecular weight markers are indicated on the left.

We took a non-biased proteomics approach to identify proteins that adsorb selectively to pooled infection IgG, and not to pooled control IgG (Figure 2A). Each serum pool contained four individual sera of patients clinically defined as having an infection with *T. b. gambiense* or as being uninfected. IgG fractions were purified from the pooled sera by affinity chromatography on protein G and then immobilised to cyanogen bromide-activated Sepharose beads. Equal amounts of infection and control IgG-Sepharose were incubated with equal amounts of *T. b. brucei* detergent cell lysate. Proteins that bound to the IgG columns were eluted by low pH, precipitated with cold ethanol, dissolved in SDS-sample buffer, reduced, separated by SDS-PAGE and stained with colloidal Coomassie blue (Figure 2B). More protein was seen in the eluate from the infection IgG column, consistent with infection-specific anti-trypanosome immune responses. Equivalent sections were cut out from the infection and control lanes, as indicated (Figure 2B). The excised gel pieces underwent in-gel S-alkylation and tryptic digestion, and the tryptic peptides were analysed by LC-MS/MS. Mascot software matched the peptide spectra to proteins in the *T. b. brucei* predicted protein database and scored the quality of the identifications. Lists of the proteins retained by infection IgG-Sepharose and control IgG-Sepharose were compared in each gel section. Twenty-four proteins with a MASCOT protein score above 50 were found uniquely in the infection IgG eluate and these are described in (Table 1). Several of the infection-specific proteins were defined as ‘hypothetical’, but other hits included known cell surface proteins, such as: Invariant Surface Glycoprotein (ISG) 75, ISG65, ISG64, Gene Related to Expression Site Associated Gene (GRESAG) 4, and the transferrin receptor subunits ESAG 6 and 7. As a starting point, the proteins with high MASCOT scores were prioritised. The rationale for this selection was that, by using an excess of trypanosome lysate in the affinity purification step, the amount of an eluted antigen should reflect, to a first approximation, the relative amount of antigen-specific immobilised IgG. The latter should, in turn, correspond to the immune response to that antigen in infected patients. Using this criterion, the protein antigens selected for study were ISG75, ESAG7, GRESAG4, ISG65, ISG64 and ESAG6 (Table 1). Next, we looked into the likely ease of protein expression of these antigens in *E. coli*. At this stage, we de-selected ESAG6 and ESAG7 because they form a heterodimer (adding the complication of dual expression) and because successful (but low level) protein expression has only been reported in a eukaryotic baculovirus expression system [47]. On the other hand, *E. coli* recombinant expression of domains of ISG75, ISG65 and ISG64 had either been reported in the literature [48] or were known to the authors (Mark Carrington, unpublished data). Consequently, we selected all three ISGs for protein expression trials. Finally, we performed expression trials on the predicted extracellular domain of GRESAG4, for which there was no literature precedent.

**Table 1:** Trypanosome proteins selectively recognised by *T. b. gambiense* infection IgG.

MASCOT score	Protein description	Protein/Gene ID	Mass	Peptides matched
**2796**	75 kDa Invariant Surface Glycoprotein (ISG)	Uniref100_Q26769	58591	100
**1456**	Gene related to Expression site-associated gene (GRESAG) 4	Tb927.7.7530	137920	67
**1152**	Expression site-associated gene (ESAG) 7	Tb427 telo10 v1 145	38433	31
**1098**	65 kDa ISG	Uniref100_Q26712	48192	39
**582**	64 kDa ISG	Tb927.5.1410	46867	23
**510**	Polyubiquitin	Tb11.01.1680	76556	10
**368**	Hypothetical protein 3020	Tb927.6.3020	32198	8
**256**	ESAG6	Uniref100_Q8WPU1	44221	10
**235**	ESAG2	Tb927.1.4890	53686	7
**220**	Flagellar calcium-binding protein TB-17	UniRef100_P17882	25477	6
**209**	Hypothetical protein 0210	Uniref100_Q386P9	51028	3
**202**	Hypothetical protein 2120	Tb927.7.2120	46341	6
**185**	Phosphoribosylpyrophosphate synthetase,	Tb10.6k15.0970	40452	7
**156**	ESAG11	Tb927.1.4900	32032	5
**140**	Hypothetical protein 4180	Tb927.6.4180	16317	2
**121**	ESAG3	UniRef100_Q8WPR9	42744	5
**89**	Gp63-3 surface protease homology	Uniref100_Q4FKH2	70254	2
**82**	Hypothetical protein 1300	Tb927.3.1300	46343	2
**70**	RNA binding protein (RBP29)	Tb10.61.3200	41052	5
**70**	Hypothetical protein 2570	Tb927.7.2570	52912	2
**59**	Flagellum-adhesion glycoprotein	Tb927.8.4060	64947	1
**56**	Hypothetical protein 4300	Tb11.02.4300	48868	2
**56**	Hypothetical protein 1910	Tb11.02.1910	36953	2
**52**	Hypothetical protein 2100	Tb927.5.2100	49921	2

### Initial ELISA screens with pooled sera

**Figure 3:**
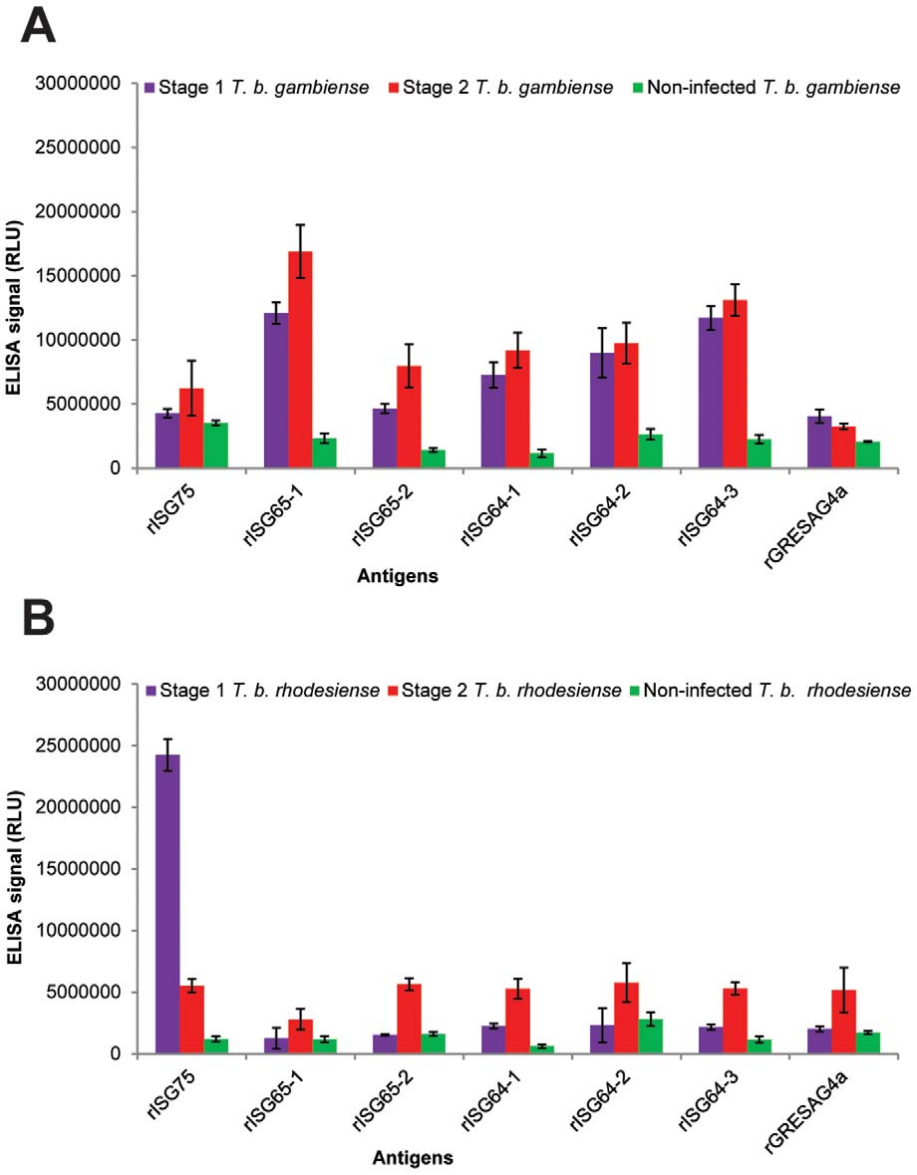
ELISA results with pooled human sera. (A) Pooled human sera representing stage 1 *T. b. gambiense* infections (pool of 10 sera), stage 2 *T. b. gambiense* infections (pool of 40 sera) and matched uninfected controls (pool of 50 sera) were diluted 1∶1000 and used in triplicate on ELISA plates coated with the rISG75, rISG65-1, rISG65-2, rISG64-1, rISG64-2, rISG64-3 and rGRESAG4a recombinant proteins described in (Figure 2). The mean ELISA signals ± SEM are plotted against the recombinant protein used in the ELISA. (B) As panel A but using pooled human sera representing stage 1 *T. b. rhodesiense* infections (pool of 5 sera), stage 2 *T. b. rhodesiense* infections (pool of 20 sera) and matched uninfected controls (pool of 25 sera).

The selected purified recombinant trypanosome proteins, see Supporting Information (Figure S1), were used to prepare ELISA plates, as described in Experimental Procedures, and these were screened against various pooled human sera. These pools were derived from the 145 individual serum samples provided by the WHO Human African Trypanosomiasis specimen bank. The pooled sera were for stage 1 *T. b. gambiense* patients (n = 10), stage 2 *T. b. gambiense* patients (n = 40) and matched uninfected patients (n = 50); and from stage 1 *T. b. rhodesiense* patients (n = 5), stage 2 *T. b. rhodesiense* patients (n = 20) and matched uninfected patients (n = 20). The results indicated that both stage 1 and stage 2 *T. b. gambiense* infection sera have significant antibody titres against all of the rISG64 and rISG65 proteins, compared to pooled non-infection sera (Figure 3A), whereas infection sera titres against rISG75 and GRESAG4a were much closer to those for the control sera. The best performing recombinant protein was ISG65-1, which had the highest infection to control signal. For the *T. b. rhodesiense* pooled sera, the signals were generally significantly lower, with the stage 2 pooled sera giving a significantly higher signal than the stage 1 pooled sera. There was one exception to this; the *T. b. rhodesiense* stage 1 pool had the highest antibody titre against rISG75 (Figure 3B). However, as will be described later, the rISG75 result was due to a very high antibody titre in a single individual. From these results, all the rISG proteins were taken forward and screened against the individual sera but GRESAG4a (rG4a) was abandoned at this stage because it had poor infection versus non-infection discrimination.

### ELISA screens with individual sera

**Figure 4:**
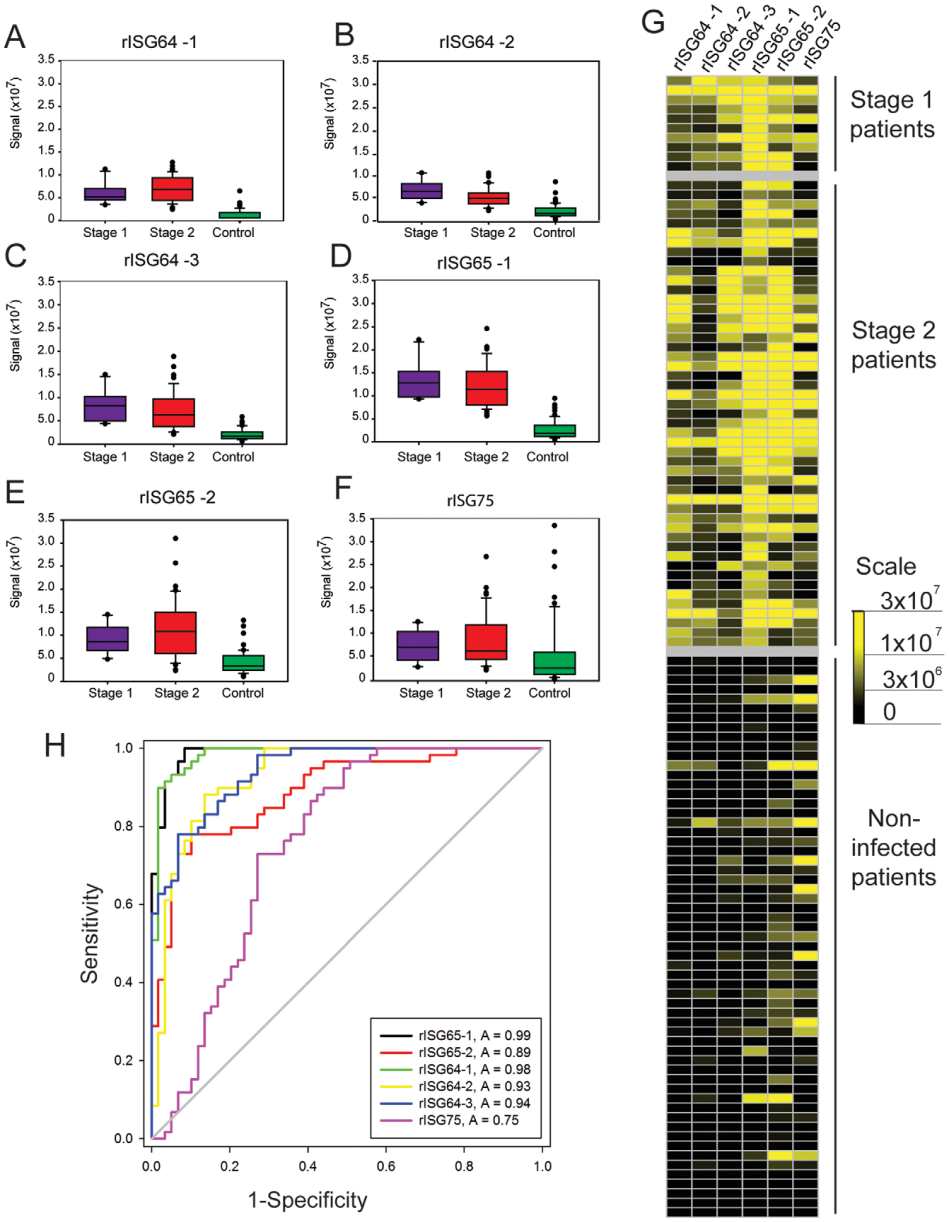
ELISA results using individual *T. b. gambiense* infection and matched control sera. (A–F) Box plots (generated by Cleveland method) represent the 25^th^ percentile to the 75^th^ percentile boundaries in the box with the median line within the box, the whiskers indicate the 10^th^ and 90^th^ percentiles. The box plots show ELISA signals for each recombinant protein ELISA plate: (A) rISG64-1, (B) rISG64-2, (C) rISG64-3, (D) rISG65-1, (E) rISG65-2 and (F) rISG75) tested against individual sera diluted 1∶1000 from stage 1 *T. b. gambiense* infections (n = 10), stage 2 *T. b. gambiense* infections (n = 40) and matched uninfected controls (n = 50). (G) Heat maps of the same data for the individual sera versus the recombinant protein ELISA plates. (H) Receiver operating characteristics (ROC) plots of the same data. The output statistics for sensitivity and specificity are shown in (Table 2).

**Figure 5:**
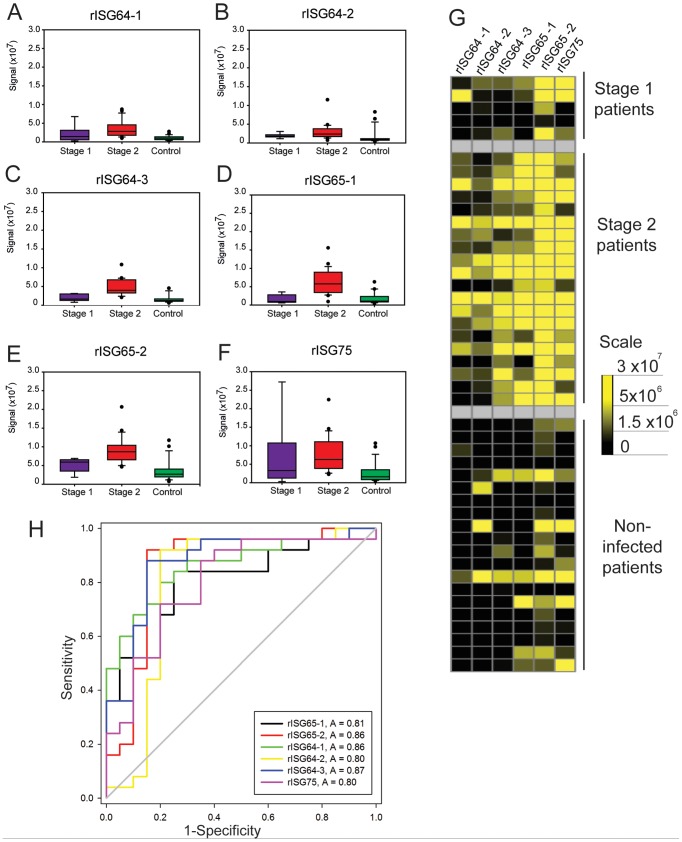
ELISA results using individual *T. b. rhodesiense* infection and matched control sera. (A–F) Box plots (generated by Cleveland method) represent the 25^th^ percentile to the 75^th^ percentile boundaries in the box with the median line within the box, the whiskers indicate the 10^th^ and 90^th^ percentiles. The box plots represent the ELISA signals for each recombinant protein ELISA plate: (A) rISG64-1, (B) rISG64-2, (C) rISG64-3, (D) rISG65-1, (E) rISG65-2 and (F) rISG75) tested against individual sera diluted 1∶1000 from stage 1 *T. b. rhodesiense* infections (n = 5), stage 2 *T. b. rhodesiense* infections (n = 20) and matched uninfected controls (n = 20). (G) Heat maps of the same data for the individual sera versus the recombinant protein ELISA plates. (H) Receiver operating characteristics (ROC) plots of the same data. The output statistics for sensitivity and specificity are shown in (Table 2).

Recombinant protein ELISA plates that performed well in the pooled sera ELISAs were further screened against all of the individual sera. These antigens included three rISG64 proteins, two rISG65 proteins and one rISG75 protein. In this case, a total of 163 individual serum samples (145 from the WHO HAT specimen bank and 18 from the Institue of Tropical Medicine, Antwerp) were diluted and applied in triplicate to wells coated with single recombinant proteins. *T. b. gambiense* and *T. b. rhodesiense* patient sera ELISA results were analysed separately (Figure 4) and (Figure 5), respectively. The data are shown as box plots for each different recombinant antigen ELISA plate (Figures 4A and 5A) to provide a visualisation the range of antibody titres and the heat maps provide a different view of the same data (Figures 4B and 5B). Both views suggest that rISG65 proteins provide the highest detection sensitivity whereas the rISG64-1 may provide slightly greater specificity. The rISG75 protein did not perform as well as the rISG65 or rISG64 proteins by both criteria and, indeed, only the stage 2 sera had statistically significant levels of IgG to rISG75-1 compared to controls (Q = 4.616, P = <0.05). Dunn's post-hoc tests (not shown) demonstrated that, whereas there are significantly higher levels of anti-rISG64 and anti-rISG65 IgG antibodies in both stage 1 and stage 2 sera compared to uninfected controls, there is no statistically significant difference between the stage 1 and stage 2 groups. In other words, relative immunoreactivity to rISG64 or rISG65 antigens cannot be used to stage of the disease. Formal sensitivity (i.e., the proportion of correct positive results) and specificity (i.e., the proportion of correct negative results) parameters for each test were calculated by ROC curve analysis (Figure 4C and 5C) and are collated in (Table 2). The recombinant antigens that best discriminated between *T. b. gambiense* infected and control patients by ELISA were rISG65-1 and rISG64-1, which had areas under the ROC curve of 0.99 and 0.98 respectively (Figure 4C). The rISG65-1 ELISA antigen had sensitivity of 96.6% (with a 95% Confidence Interval (CI) of 88.3 to 99.6%) and specificity of 93.2% (95% CI of 83.5 to 98.1%), whereas sensitivity and specificity of rISG64-1 antigen was 93.2% (95% CI of 83.5 to 98.1%) and 94.9% (95% CI of 85.9 to 98.9%), respectively (Table 2).

**Table 2:** Sensitivities and specificities of the recombinant ISG ELISAs.

Antigen	Infectious species	Sensitivity	95% CI	Specificity	95% CI
rISG65-1	*T. b. gambiense*	96.6	88.3 to 99.6	93.2	83.5 to 98.1
	*T. b. rhodesiense*	84	63.9 to 95.5	75	50.9 to 91.3
rISG65-2	*T. b. gambiense*	83.1	71 to 91.6	72.9	59.7 to 83.6
	*T. b. rhodesiense*	92	74 to 99	85	62.1 to 97
rISG64-1	*T. b. gambiense*	93.2	83.5 to 98.1	94.9	85.9 to 98.9
	*T. b. rhodesiense*	84	63.9 to 95.5	75	50.9 to 91.3
rISG64-2	*T. b. gambiense*	88.1	77 to 95.1	86.4	75 to 94
	*T. b. rhodesiense*	92	74 to 99	80	56.3 to 94.3
rISG64-3	*T. b. gambiense*	86.4	75 to 94	83.1	71 to 91.6
	*T. b. rhodesiense*	88	68.8 to 97.5	85	62.1 to 97
rISG75-1	*T. b. gambiense*	72.9	59.7 to 83.6	72.9	59.7 to 83.6
	*T. b. rhodesiense*	72	50.6 to 87.9	75	50.9 to 91.3

It was more difficult to find a recombinant protein antigen that reliably discriminated *T. b. rhodesiense* infected patient sera from non-infected sera. The box plots, heat maps (Figure 5A and B) and Dunn's post hoc analyses (not shown) all indicate that, whereas the stage 2 sera show statistically significant immunoreactivity to all the antigens compared to controls, the immunoreactivities of the stage 1 sera are not statistically significant. rISG65-2 was the most sensitive at identifying *T. b. rhodesiense* infection sera 92% (95%, CI of 74 to 99%), but at a cost to specificity 85% (95%, CI 62.1 to 97%) (Figure 5C and Table 2). As mentioned above, the pooled sera ELISA experiments had indicted that stage 1 *T. b. rhodesiense* infection sera might have high antibody titres towards rISG75. However this proved not to be the case and was due to a single serum sample with a very high anti-rISG75 titre.

### Lateral flow prototype development to detect *T. b. gambiense* infections

**Figure 6:**
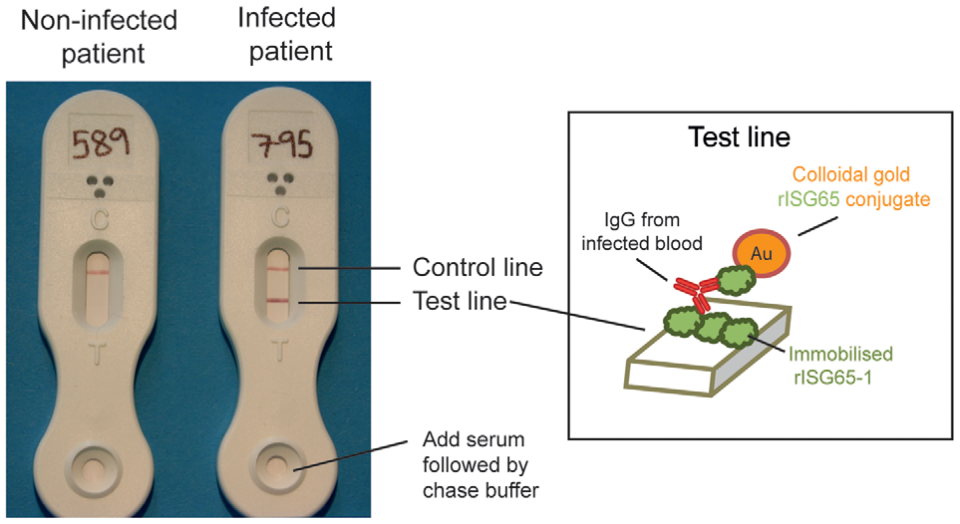
Prototype lateral flow device for detecting antibodies to rISG65-1 protein. Representative results using serum samples from a matched uninfected patient (left) and a stage 2 *T. b. gambiense* infected patient (right). The visual scores for these test lines were 0 and 5, respectively, and the CAMAG densitometry measurements were 24.2 and 597.4, respectively. The inset shows the principle of detection, with patient antibody to ISG65 forming a bridge between rISG56-1 immobilised on the nitrocellulose strip and the colloidal gold-coupled rISG65-1 picked up from the sample pad.

Based on the ROC curve analyses of the performances of the ELISA plates, we selected rISG65-1 (ROC curve area 0.99 for *T. b.gambiense* sera) for development of a lateral flow prototype. Purified rISG65-1 was supplied to BBInternational (Dundee, www.bbigold.com) a company that specialises in lateral flow technology. The lateral flow approach that was utilised is illustrated in (Figure 6). Thus, rISG65-1 was both immobilised in a band on a nitrocellulose membrane and coupled to colloidal gold that was then localised in the conjugate pad. When the sera and chase buffer are applied to the sample pad, the rISG65-colloidal gold conjugate is resuspended. The absorbent pad at the top of the lateral flow device draws the liquid across the nitrocellulose membrane. During this time, any anti-rISG65 antibody in the serum binds to the rISG65-gold conjugate and when the antibodies reach the rISG65 test band, one Fab arm of the IgG binds to the immobilised rISG65 while the other Fab domain bridges to the rISG65-gold-conjugate. Accumulation of this specific antibody sandwich generates a visible test line. The control line is an internal positive control for the lateral flow test and does not relate to the infection status of the patient but indicates successful test flow. The final reading of this test should be as follows; the appearance of only a control line (upper band) indicates non-infected sera, whereas, the appearance of two lines, a control and test line (upper & lower bands) indicates infected sera, examples are shown in (Figure 6). Absence of a control line (upper band) indicates an invalid test, irrespective of the appearance of the test line and the test should be repeated.

### Prototype validation

**Figure 7:**
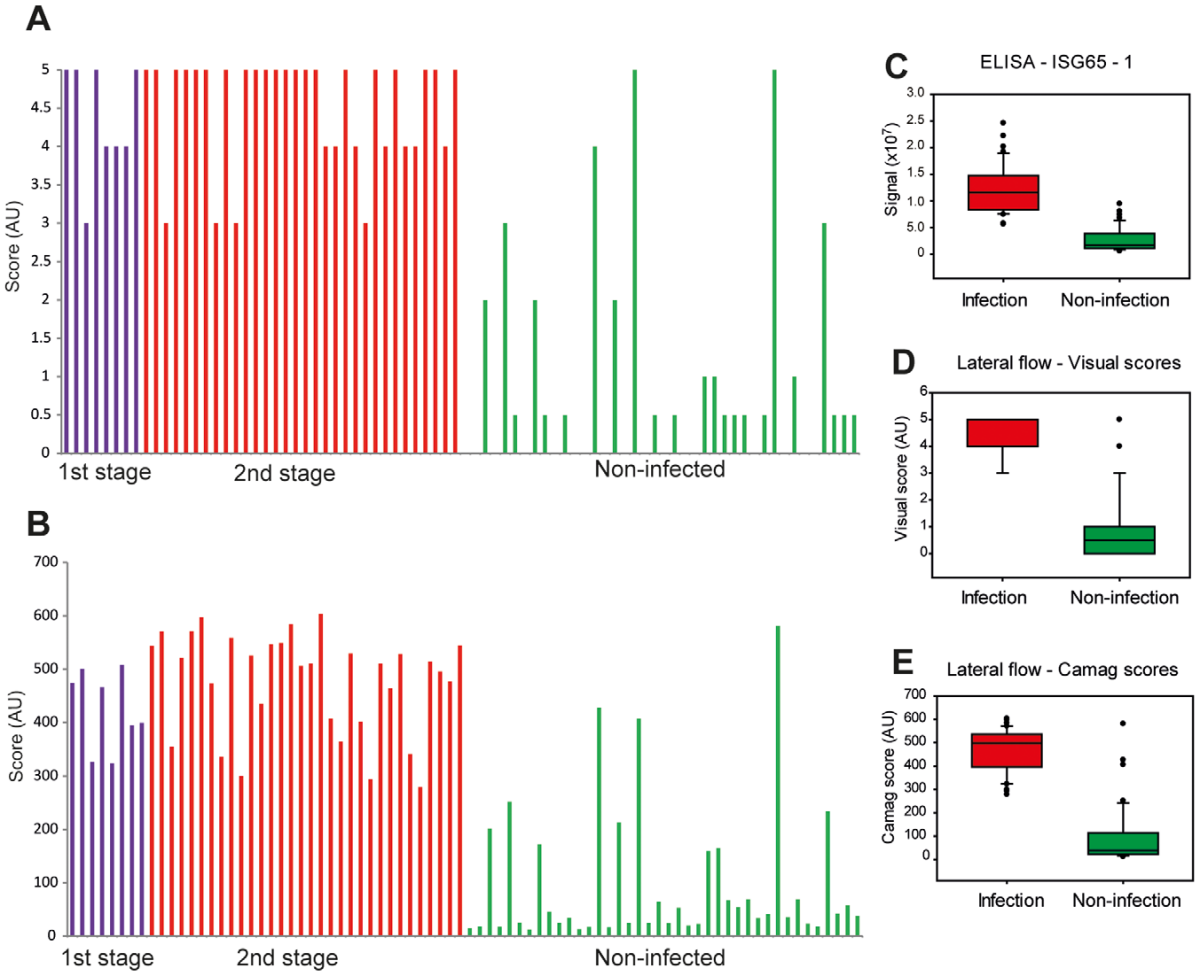
Performance of the prototype lateral flow device in a blinded study with eighty randomised serum samples. (A) Visual scores of test line density from rISG65-1 prototype lateral flow devices (scored in increments of 1 from 0 to 5, with very faint test line shadows represented as 0.5) are plotted against the subsequently decoded patient status (stage 1 *T. b. gambiense* infections (n = 8), stage 2 *T. b. gambiense* infections (n = 32) and matched uninfected controls (n = 40). (B) The same test strips were removed from the devices and scanned by CAMAG densitometer. The data are plotted directly below the results for the visual scores for the same samples. The R^2^ of a scatter plot was 0.96, showing very good correlation between visual score and CAMAG reading. (C–E) Box plots of the results for the same serum samples analysed by (C) rISG65-1 ELISA, (D) rISG65-1 lateral flow prototype with visual scoring and (E) rISG65-1 lateral flow prototype with CAMAG scanner scoring.

Eighty randomised and coded WHO ‘test’ *T. b. gambiense* sera, comprising forty infected and forty non-infected sera were applied to the lateral flow prototypes. Each serum sample (5 µl) was diluted with 15 µl of PBS and applied to a lateral flow device sample pad. Within about 30 s, 80 µl of chase buffer was added and the test was left for 30 min, at which point a visual score was recorded. The sample pads were removed to prevent back flow and the visual scores were decoded (Figure 7). Sensitivity and specificity were calculated by ROC curve analysis, and for visual scores a cut off of 2.5 gave 100% sensitivity (95% CI of 91.1 to 100) and 87.5% specificity (95% CI of 73.2 to 95.8%). An analysis of the test lines was also carried out using a densitometer, where an arbitrary cut off at 265.6 RU gave 100% sensitivity (95% CI of 91.2 to 100%) and 92.5% specificity (95% of CI 79.6 to 98.4%) (Table 3) indicating there is potential for separation between infection and non-infected individual scores. Principally the end user will interpret the results visually therefore further optimisation of the test line will be necessary to reduce false positive results due to non-specific binding. A checklist, Supporting Information (Table S2), and flow diagram, Supporting Information (Figure S2), are provided according to the STAndards for the Reporting of Diagnostic accuracy studies (STARD) guidelines.

**Table 3:** Sensitivity and specificity values.

Test/Antigen	Sensitivity	Specificity
CATT	87–98%^1^	93–95%^1^
rISG65-1 (ELISA data)	95% (95% CI, 88 to 100%)	93% (95% CI, 83.5 to 98%)
rISG65-1 lateral flow (visual assesment)	88% (95% CI, 73 to 96%)	93% (95% CI, 79.6 to 98%)
rISG65-1 lateral flow (Camag scanner)	100% (95% CI, 91 to 100%)	93% (95% CI, 79.6 to 98%)

1 Taken from [31] for the CAAT test. The values for the rISG65-1 ELISA and lateral flow device using *T. b. gambiense* infection and control sera were calculated from the data presented in this paper.

## Discussion

The overall goal of this project was to develop an immunodiagnostic lateral flow prototype for human African trypanosomiasis that might be developed into a field-based device to replace the CATT screening tool. To do this, we needed to identify potential diagnostic antigen candidates, investigate whether they could be adequately expressed and purified and assess their diagnostic potential with patient sera. We took an unbiased proteomics approach to identify more than twenty potential diagnostic protein antigens, several of which were known cell-surface glycoproteins. This list was filtered pragmatically; first, the proteins with high proteomic MASCOT scores, generally synonymous with their abundance, were selected because, by using an excess of trypanosome lysate in the affinity purification step, the amount of an eluted antigen should reflect the relative amount of antigen-specific IgG in infection sera. The latter should, in turn, correspond to the immune response to that antigen in infected patients. From this list we eliminated ESAG6, since it is known to form a heterodimer with ESAG7 and is, therefore, relatively complicated to express [46]. Our attempts to express parts of the extracellular domain of GRESG4 in *E. coli* were not very successful, although we were able to isolate the A-domain fused to GST. However, this G4a construct and the ISG75 protein construct did not perform well in the ELISA studies and were removed from this study. Nevertheless, these antigens should not be ignored for diagnostic development as they may simply have been miss-folded in the absence of endoplasmic reticulum folding and quality control components [49]. Indeed, recombinant ISG75 has been shown to have diagnostic potential for *T. b. brucei* animal infections [47]. Future expression attempts might include bacterial expression systems that target recombinant proteins into the periplasmic space [49] and/or eukaryotic expression systems such as insect cells and *Pichia pastoris*.

Our data on the diagnostic potential of ISGs 64, 65 and 75 for detecting *T. b. rhodesiense* infections were somewhat hampered by the small number of sera available for testing. Nevertheless, it is clear from the ELISA data that IgG antibody responses to these antigens are lower than in *T. b. gambiense* infections. Further, the IgG responses are particularly low in stage 1 *T. b. rhodesiense* patient sera. This may be due to the differing nature and speed of progression of the infections; *T. b. rhodesiense* infections are usually acute and progress faster whereas *T. b. gambiense* infections are chronic and progress over months or years [50], which could in turn lead to a greater amount and diversity of antibodies present in these sera. A previous study also struggled to identify diagnostic antigens for *T. b. rhodesiense* infections [51]. A good approach may be to repeat the procedures described here using immobilised IgG from *T. b. rhodesiense* patient sera.

Further research is also required to measure the half-life of antibodies in patients after they have been treated for HAT, as persistent antibodies may lead to false positives. It has been described that antibodies can persist up to 3 years post cure, however it is not known which class of antibodies persist or which antigens they recognise [52]. Ideally, a longitudinal study could be carried out to gain a greater insight into this and how it could affect the diagnostic potential of any future lateral flow test relying on antibodies [32]. Lateral flow tests, whilst having limitations, could potentially be more suitable for use in the field because of their stability and the fact that they can be used by non-specialists [53].

In summary, we report here the selection of ISG65-1 as a potential diagnostic antigen for *T. b. gambiense* infections and its performance in both conventional ELISA and prototype lateral flow device assays looks promising. The performance of the prototype ISG65 lateral flow device encourages us to further develop and optimize it, perhaps adding an additional antigen or antigens to improve sensitivity and specificity, while aiming for a production cost of <US$1 per unit.

## Supporting Information

Figure S1 **Coomassie blue stained SDS-PAGE gels of the purified recombinant** ***T. brucei*** **protein domains.**
(DOC)

Figure S2 **STARD flow chart.** STAndards for the Reporting of Diagnostic accuracy studies (STARD) description of the experimental design to calculate sensitivity and specificity of the lateral flow device.
(PDF)

Table S1 **Source of genomic DNA and PCR primers used to clone the trypanosome protein domains and the optimized protein expression conditions used for recombinant protein production in** ***E. coli*** **.**
(DOC)

Table S2 **STARD checklist.** STAndards for the Reporting of Diagnostic accuracy studies (STARD) checklist for reporting of studies of diagnostic accuracy.
(DOC)

Text S1 **Details of ISG and GRESAG protein domain expression and purification.**
(DOC)
