# Supplementary material for: Proteomic Selection of Immunodiagnostic Antigens for Human African Trypanosomiasis and Generation of a Prototype Lateral Flow Immunodiagnostic Device
Source: PLoS Negl Trop Dis. 2013 Feb 28;7(2):e2087. doi: 10.1371/journal.pntd.0002087 (PMC3584999; doi:10.1371/journal.pntd.0002087)
Supplement: Figure S1 — Coomassie blue stained SDS-PAGE gels of the purified recombinant T. brucei protein domains. (DOC) [file pntd.0002087.s001.doc]

**
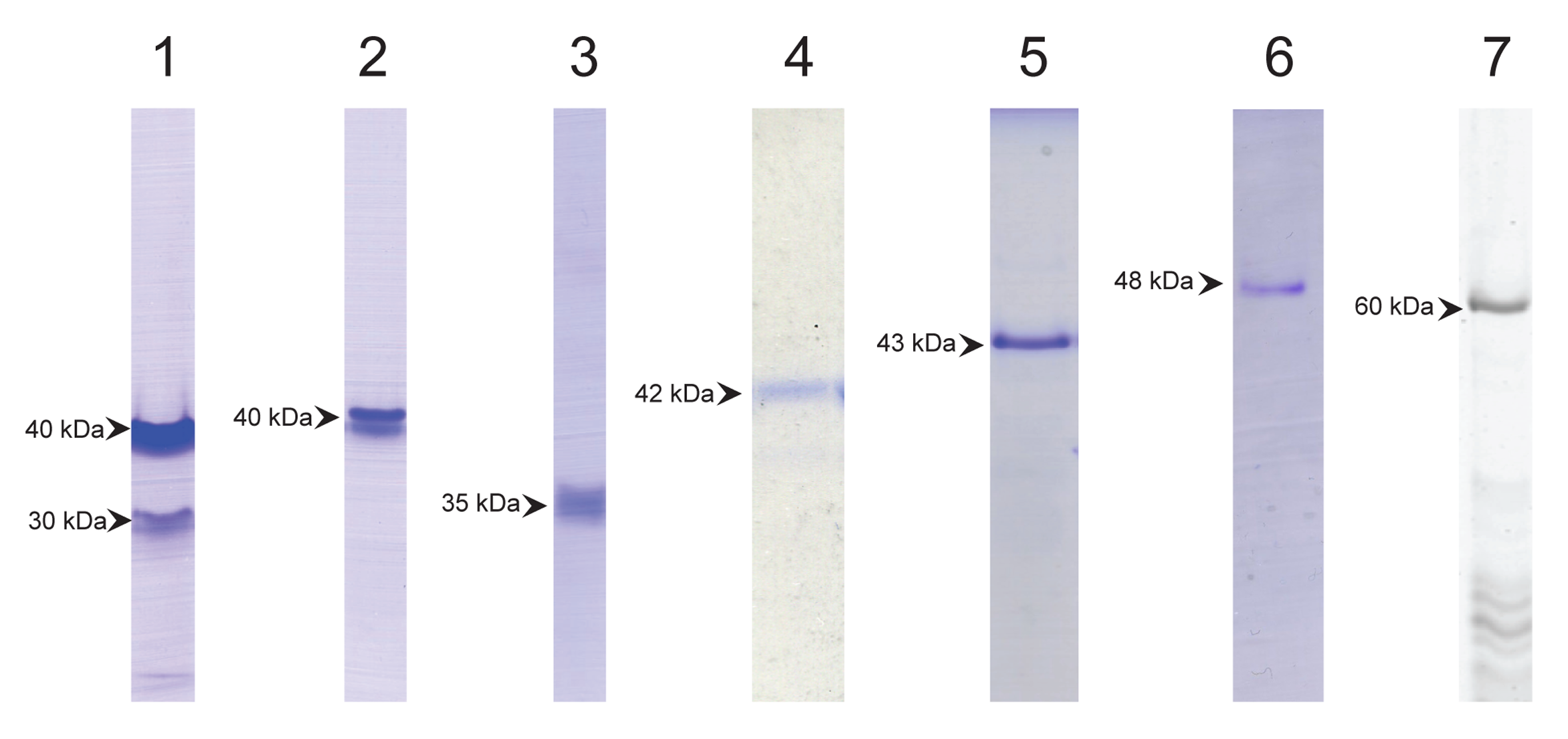
**

**Figure S1.** Coomassie blue stained SDS-PAGE gels of the purified recombinant *T. brucei* protein domains. Lane 1: rISG64-1; Lane 2: rISG64-2; Lane 3: rISG64-3; Lane 4: rISG65-1; Lane: 5 rISG65-2; Lane 6: rISG75-1 and Lane 7: GST-G4a. In all cases, the identities of the arrowed bands were confirmed by proteomics. Note: In the case of rISG64-1 (lane 1) a proteolysis product of 31 kDa was also present and both proteins were used to coat ELISA plates.
