# Supplementary figures and images for: Proteomic Selection of Immunodiagnostic Antigens for Human African Trypanosomiasis and Generation of a Prototype Lateral Flow Immunodiagnostic Device
Source: PLoS Negl Trop Dis. 2013 Feb 28;7(2):e2087. doi: 10.1371/journal.pntd.0002087 (PMC3584999; doi:10.1371/journal.pntd.0002087)

Figure S2

## Lateral flow test flow chart

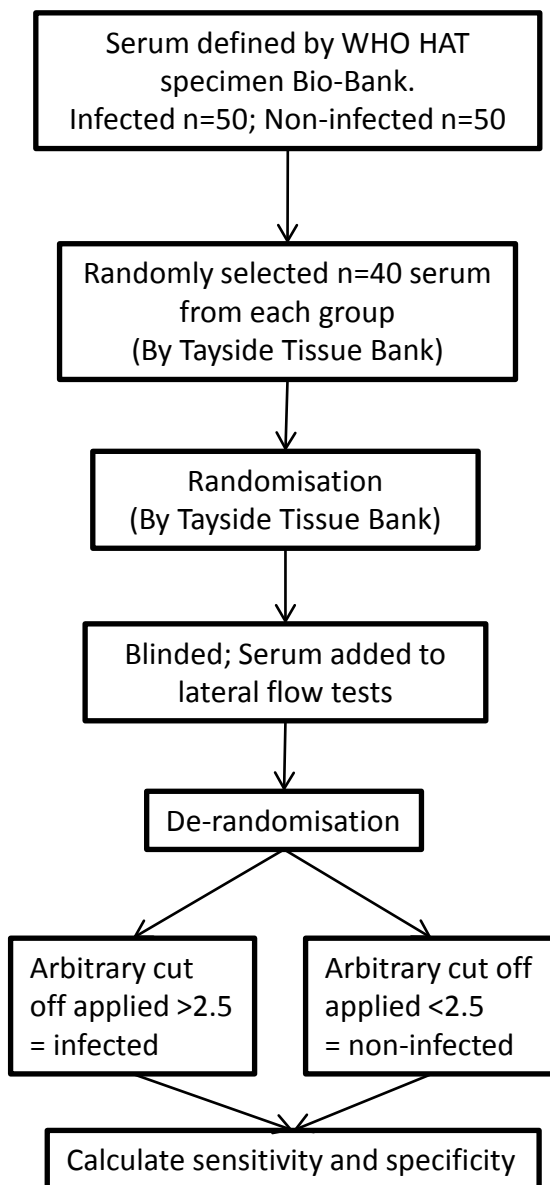

Supplement: Figure S2 — STARD flow chart. STAndards for the Reporting of Diagnostic accuracy studies (STARD) description of the experimental design to calculate sensitivity and specificity of the lateral flow device. (PDF) [file pntd.0002087.s002.pdf]
