# Supplementary material for: Proteomic Selection of Immunodiagnostic Antigens for Human African Trypanosomiasis and Generation of a Prototype Lateral Flow Immunodiagnostic Device
Source: PLoS Negl Trop Dis. 2013 Feb 28;7(2):e2087. doi: 10.1371/journal.pntd.0002087 (PMC3584999; doi:10.1371/journal.pntd.0002087)
Supplement: Table S1 — Source of genomic DNA and PCR primers used to clone the trypanosome protein domains and the optimized protein expression conditions used for recombinant protein production in E. coli . (DOC) [file pntd.0002087.s003.doc]

**Table S1.** Source of genomic DNA and PCR primers used to clone the trypanosome protein domains and the optimized protein expression conditions used for recombinant protein production in *E. coli.*

| **Protein** | **Source of *T. b. brucei* genomic DNA** | **Upstream Primers (5’ to 3’)** | **Downstream primer (5’ to 3’)** | **Induction OD** | **Expression time (h)** | **Expression temperature (^o^C)** |
| --- | --- | --- | --- | --- | --- | --- |
| **rISG65-1** | EATRO1125 | CATATGGAAAATCTGTACTTCCAAGGCGAAGCTTCTAATGGTGGAGATAA | GGATCCTTATCTTCTCTGATGTCTGCTTTTTAC | 0.8 | 16 | 18 |
| **rISG65-2** | EATRO1125 | CCATGGACAAGAATTTGACGAAAG | CTCGAGTTTCTGATGTCTACGC | 0.5 | 16 | 20 |
| **rISG64-1** | Lister 427 | TATAATTACTCGAGAATGCAAAGTTGACCAAAGATGGTGCGTT | TATAATAGGATCCTTAATCACTAGTCTCCAGGAGGTCACCGAA | 0.3 | 3 | 37 |
| **rISG64-2** | EATRO1125 | CCATGGAAACAAATGAGGAAGCGAAG | CTCGAGGCTCTCCTTGATCTTACTGC | 0.3 | 3 | 37 |
| **rISG64-3** | EATRO1125 | CATATGGAAAATCTGTACTTCCAAGGCGAAACAAATGAGGAAGCGAAG | GGATCCTTACCCTCGCTGTGTGGCCTCG | 0.3 | 3 | 37 |
| **rISG75** | EATRO1125 | CATATGGAGGAGCTCTCTGTTGC | CTCGAGCTTCGTTGTCCCAATCCAGCC | 0.4 | 3 | 37 |
| **G4a (50-401) for pGEX vector** | Lister 427 | TAATTACATATGATTTCAGCAAAGGTATATGATCCTATTACTGCA | TAATTACTCGAGTTAGTCACCAACCACAAGATCATCAATTACATAAC | 0.6 | 16 | 20 |
