# Supplementary material for: Proteomic Selection of Immunodiagnostic Antigens for Human African Trypanosomiasis and Generation of a Prototype Lateral Flow Immunodiagnostic Device
Source: PLoS Negl Trop Dis. 2013 Feb 28;7(2):e2087. doi: 10.1371/journal.pntd.0002087 (PMC3584999; doi:10.1371/journal.pntd.0002087)
Supplement: Text S1 — Details of ISG and GRESAG protein domain expression and purification. (DOC) [file pntd.0002087.s005.doc]

**Text S1**

*Details of* *ISG and GRESAG protein domain expression and purification*

The amplified ISG gene segments were cloned into various pET bacterial expression plasmids that provide a His-tag fused either to the N-terminus or C-terminus of the protein, in some cases via a TEV protease cleavage site, as indicated in (Figure 1). The plasmids were transformed into the BL21(DE3)RIPL (Stratagene) strain of *Escherichia coli*. Selected clones were grown aerobically at 37 ^o^C in 2x Bacto-Tryptone and yeast extract (TY) medium containing 50 µg/ml carbenicillin and 12.5 µg/ml chloramphenicol. The expression of recombinant proteins was induced with a final concentration of 1 mM isopropyl β-ᴅ-thiogalactopyranoside (IPTG) under conditions optimised for each one (Table S1). For the purification of the His-tagged proteins, the *E. coli* cells were harvested by centrifugation at 6000 x *g* for 15 min at 4^o^C, resuspended in lysis buffer (50 mM NaH_2_PO_4_, 300 mM NaCl, 10 mM imidazole, pH8) and lysed by French Press. Cell debris was removed by centrifugation for 15 min at 12,600 x *g*, 4^o^C and the supernatant was pre-cleared with lysis-buffer-equilibrated Sepharose 6B resin for 10 min. The Sepharose 6B was removed by centrifugation (1000 x *g* for 1 min) and the supernatant was combined with lysis-buffer-equilibrated Nickel-agarose beads for 1 h at 2 ^o^C. The beads were recovered by centrifugation (1000 x *g* for 1 min), washed with 50 ml lysis buffer and transferred to a column where the beads were further washed with 50 ml lysis buffer and with 50 ml lysis buffer containing 20 mM imidazole. Finally, the His-tagged proteins were eluted by the addition of 10 ml lysis buffer containing 250 mM imidazole. The eluted samples were dialysed against lysis buffer before being flash-frozen in liquid nitrogen for storage at -80^o^C. Samples for SDS-PAGE analysis were taken along the purification process to monitor for the presence of the overexpressed recombinant proteins and to estimate the purity of the protein in the final eluates. The SDS-PAGE analyses of the purified recombinant proteins are shown in (Figure S1). The identities of the recombinant proteins were confirmed by tryptic digestion and proteomic analysis of the indicated bands. Bio-Rad protein assay and absorbance at 280 nm were used to determine protein concentrations. The His-rISG65-1 protein underwent TEV proteolytic cleavage by adding recombinant His-TEV in a ratio of 10:1 (protein: TEV) (3 h at room temperature). To separate the His-TEV from the free rISG, the mixture was applied to a second (pre-equilibrated) Ni-column to capture His-TEV, *E. coli* His-rich contaminant proteins and uncleaved His-ISG65-1 protein.

Multiple constructs were designed for GRESAG4 encoding the predicted full-length extracellular domain and several small globular domains based on predictions from GLOBplot software (Figure 1). These constructs were amplified from genomic DNA using the primers described in (Table S1), cloned into TOPO-TA vector pCR2.1 and verified by DNA sequencing. The constructs were either cloned into the pET15bTEV vector, such that the proteins they encode are fused at the N-terminus to a His tag, or into a pGEX-TEV vector such that the protein is fused at its N-terminus to a glutathione S-transferase (GST) sequence via a TEV cleavage site. These plasmids were transformed into *E. coli* BL21(DE3)RIPL cells and clones were grown aerobically at 37^o^C in 2x Bacto-Tryptone and yeast extract (TY) medium containing 50 µg/ml carbenicillin and 12.5 µg/ml chloramphenicol. The expression of recombinant proteins was induced with a final concentration of 1 mM isopropyl β-ᴅ-thiogalactopyranoside (IPTG) under the conditions described in (Table S1). Only the G4a construct produced soluble protein, which was purified as follows: *E. coli* cells containing pGEX-G4a were harvested by centrifugation at 4000 x *g* for 30 min at 4^o^C. The cell pellet was resuspended in lysis buffer and cells were lysed using a French Press. The lysate was clarified by centrifugation at 30,000 x *g* for 30 min, 4^o^C. The supernatant was filtered using 0.45 µM filter and applied to a pre-equilibrated 1 ml GST-trap column (GE Healthcare). The column was washed with lysis buffer and eluted by application of a isocratic elution step (AKTA purifier, GE Healthcare) of lysis buffer containing fresh 20 mM reduced glutathione. The fractions obtained were checked by SDS-PAGE gel electrophoresis, and those containing GST-G4a were pooled and concentrated using a centrifugal concentrator (Millipore, 0.5 ml capacity with 30 kDa MW cut off) at 4^o^C (Figure S1). Attempts to remove the GST tag were unsuccessful therefore the fusion protein was used for further experiments.
